# Interplay Between the Immune and Nervous Cognitive Systems in Homeostasis and in Malaria

**DOI:** 10.7150/ijbs.82556

**Published:** 2023-06-28

**Authors:** Luciana Pereira de Sousa, Pamela Rosa-Gonçalves, Flávia Lima Ribeiro-Gomes, Cláudio Tadeu Daniel-Ribeiro

**Affiliations:** 1 Laboratório de Pesquisa em Malária, Instituto Oswaldo Cruz & Centro de Pesquisa, Diagnóstico e Treinamento em Malária (CPD-Mal) from Fundação Oswaldo Cruz (Fiocruz) and the Secretaria de Vigilância em Saúde (SVS), Ministério da Saúde, Brazil.; 2 Laboratório de Biologia, campus Duque de Caxias, Colégio Pedro II, Brazil.

**Keywords:** immune system, nervous system, homeostasis, malaria, neurocognitive impairment.

## Abstract

The immune and nervous systems can be thought of as cognitive and plastic systems, since they are both involved in cognition/recognition processes and can be architecturally and functionally modified by experience, and such changes can influence each other's functioning. The immune system can affect nervous system function depending on the nature of the immune stimuli and the pro/anti-inflammatory responses they generate. Here we consider interactions between the immune and nervous systems in homeostasis and disease, including the beneficial and deleterious effects of immune stimuli on brain function and the impact of severe and non-severe malaria parasite infections on neurocognitive and behavioral parameters in human and experimental murine malaria. We also discuss the effect of immunization on the reversal of cognitive deficits associated with experimental non-severe malaria in a model susceptible to the development of the cerebral form of the illness. Finally, we consider the possibility of using human vaccines, largely exploited as immune-prophylactics for infectious diseases, as therapeutic tools to prevent or mitigate the expression of cognitive deficits in infectious and chronic degenerative diseases.

## Introduction

Both the immune and nervous systems perform cognitive tasks (“to know and to recognize”) by mobilizing processes endowed with specificity and memory and may, therefore, be considered “cognitive systems” 1-5. Such systems organize themselves functionally and structurally, establishing new cellular connections in response to stimuli 1-3. Following an antigenic or sensory experience, organisms begin to differ in relation to their previous arrangement, not only in their experiences and skills but also in their structures 1-3. Cognitive systems can, thus, be characterized as plastic arrangements, and animals endowed with nervous and immune systems may be thought of as “cloneable organisms”, but not as “cloneable beings” 6.

The immune and nervous systems interact closely and constantly, and the influence of nervous system stimuli on the immune response, as well as the contribution of immune components to the homeostasis of cognitive function, are well documented and illustrative of this interplay 4,7-10. The evidence for the nervous system's influence on the quality and fate of immune responses will not be considered further here.

**Figure 1:**
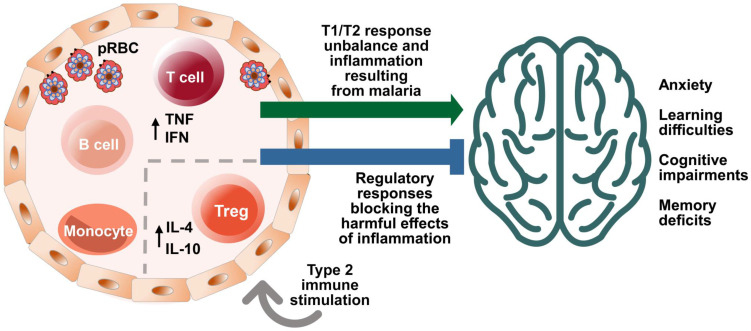
** Effects of malaria and immune system stimuli on the nervous system performance**. Immune events are involved in brain function and neurocognitive homeostasis. T cells and microglia are classically described as important for the maintenance of neurogenesis in the hippocampus, being associated with the capacity for learning and spatial memory in adulthood 7. The immune system influence is dependent upon the nature and intensity of the (activating or suppressing) stimuli it applies on the nervous system, benefitting or impairing its function. Infectious agents, such as malaria parasite, while promoting immune learning, may also exert signals perceived as disruptive to the immune response, differently from the positive properties of non-infectious stimuli, such as vaccines inducing type 2 immune responses. High levels of cytokines in the peripheral circulation and intracerebroventricular space, such as Interferon (IFN) and Tumor Necrosis Factor (TNF)**,** produced by T cells in response to red blood cell infection (pRBC) and rupture, can impair the brain function and the cognitive ability 49. On the other hand, the presence of T cells that produce anti-inflammatory cytokines, such as the interleukin 4 (IL-4), has been associated with improved cognitive ability and learning through the induction of brain-derived neurotrophic factor (BDNF) expression by astrocytes 12. Neurocognitive impairment (learning and memory deficits and anxiety-like behavior) caused by a single episode of non-severe experimental murine malaria can be attenuated or even avoided by exposure to anti-inflammatory immune stimuli that included the largely used diphtheria tetanus human vaccine (Td) 123. Increased numbers of regulatory T cells (Treg) in the spleen and interleukin 10 (IL-10) levels in the peripheral circulation observed in experimental studies may be involved in such effect 24.

The immune system acquires new experiences (immunological learning) through contact with infectious or non-infectious foreign agents and their antigens or through active immunization 11. Different stimulus strategies and immune responses can affect central nervous system (CNS) function in different ways (Fig. 1) 5,9,12-15.

T cells and the proteins they produce play a critical role in regulating the immune response and influencing brain function 9. The impact of immune stimuli on the nervous system can vary depending on their nature and context 9. In general, proinflammatory immune responses are believed to have a negative effect on cognition, while anti-inflammatory immune responses may be protective or beneficial 9,12,13,15. For example, cytokines, which function like neurotransmitters, can modulate synaptic activity and affect cognitive ability in the *quad-partite* synapse model (involving pre-synaptic and post-synaptic neurons, astrocytes and microglia). Furthermore, T cells can protect the nervous system from neuronal degeneration, and the IL-4 cytokine has been shown to maintain the M2 profile of meningeal myeloid cells and regulate astrocytic activity in brain-derived neurotrophic factor (BDNF) production, thus improving cognitive performance related to learning and memory 9,12,13,15.

Infectious diseases may manipulate or disrupt the immune response and have been associated with neurocognitive deficits 16. Malaria, toxoplasmosis, leishmaniasis and other protozoan diseases can cause neurocognitive impairment and/or anxiety-like behavior in humans and/or in murine experimental models 17-24.

Malaria, a disease caused by parasites of the genus *Plasmodium* and transmitted through the bite of female *Anopheles* mosquitoes, is one of the main public health problems in the world 25. Eight species of *Plasmodium* are now known to cause disease in man: *P. malariae*, *P. vivax*, *P. falciparum*, *P. ovale curtisi* and *P. ovale wallikeri* 26, *P. knowlesi* 27 and, as recently demonstrated, *P. cynomolgi* 28 and *P. simium* 29. Although the latter three are to be classified as non-human primate parasites that can cause zoonotic infections in humans, it became recently clear that other species of *Plasmodium* can cause malaria in humans 30,31.

Malaria can cause cognitive-behavioral impairment, especially, but not exclusively, in its brain-injurious form (cerebral malaria, CM), in humans and mice 17,22-24,32,33.

This review focuses on the effect of the immune system on the nervous system and the influence of malaria parasite infection on neurocognitive performance. We consider CM as well as the non-severe form of the disease in both humans and mice.

## Interaction between the immune and nervous cognitive systems in homeostasis

Both the immune and the nervous systems exhibit innate and adaptive (learned) responses. Innate responses of the nervous system are genetically controlled and linked to the physical structure of the brain and the CNS, while adaptive responses result from the interaction between experience and neuronal plasticity. Innate behavior refers to condition (structure, vocation and instinct), while adaptive behavior refers to learning resulting from experiences and living, which can include different types of learned behavior, such as habituation, imprinting, and classical and operant conditioning 34,35. Both rely on neural circuits (that can be "overlapping", if they share some common neurons or pathways; "shared", if they involve the same neurons or pathways between different behaviors or processes; or "evolutionarily common", if they have been conserved across different species over time) being excited by intrinsic and extrinsic stimuli 36. Therefore, we can consider that attributes traditionally considered characteristic of the immune system may also be true for the nervous system, such as the ability to change its cellular and biomolecular structures and organizations as they are stimulated 36.

In addition to being strategically and operationally similar, the immune and nervous systems are closely linked both in structural and functional terms, sharing molecular mechanisms and cellular ligands and receptors 37.

The fundamentals of “classical neuroimmunology” such as the existence of neuropeptide receptors on the lymphocyte membrane and of cytokine receptors on neurons, microglia and astrocytes, have been extensively described 14,38-41. More recently it has been shown that, in addition to T lymphocytes, immune cells such as B lymphocytes, macrophages and dendritic cells express specific neurotransmitter receptors that affect the function of these cells upon receipt of information from the nervous system 37.

It is not surprising, therefore, that immune events influence brain function and neurocognitive homeostasis. CD4 T lymphocytes and microglia are important for the maintenance of neurogenesis in the hippocampus and are associated with the capacity for learning and spatial memory in adulthood 7.

More recently, a “brain super autoantigens theory” has been proposed, postulating autoimmunity as a physiological process naturally involved in brain function. According to this theory, the driving agents of cognitive evolution are pro-cognitive T cells reactive to CNS autoantigens 10. Neurons expressing specific immunogenic antigens stimulate a repertoire of cognitive-promoting autoimmune T cells, and, as a result, the generation and diversity of brain autoimmune T cells can drive selective pressure on the neural genes that compile brain super autoantigens. Such reciprocal pressure stimulates neurocognition - formed by neurons, synapses and non-neuronal cells (such as glial cells) - orchestrating cognitive competence and resulting in the coevolution of both systems 10.

The reversal of cognitive deficits in adult mice and neonate mice lacking lymphocytes by adoptive transfer of T and lymphoid cells, respectively, illustrates the importance of adaptive immunity in brain maturity and cognitive ability 42,43. Likewise, acute and peripheral T cell depletion, induced by the administration of FTY720 [a compound that promotes the internalization of the sphingosine 1-phosphate receptor 1 and, consequently, the sequestration of thymocytes in lymph nodes 44,45, results in impaired learning and memory in mice 12.

The nervous system was considered for many years an immune privileged site, with the exception of microglia, any immune signal in the parenchyma was considered to be the hallmark of pathology 4. When T cells were first suggested as modulators of cognitive performance, questions were raised concerning the mechanisms that drive them to the brain compartment 46. It is possible that T cells penetrate the CNS parenchyma, but rarely and for very short periods of time 46. They may also affect the CNS through the release of cytokines into the bloodstream, penetrating the blood-brain barrier (BBB) by volume diffusion, transport systems 47 or CD4 T cells trafficking across the choroid plexus and meninges 8. Another pathway proposed for the entry of immune cells and their products into the brain is *via* the circumventricular organs (CVOs) 48,49. Brain-resident CD4 T cells have been identified in mice and humans and may be required for the maturation of microglia, proper synaptic pruning and behavior 50.

It is estimated that human cerebrospinal fluid (CSF) contains up to 500,000 T cells, the majority being memory cells (CD45RO^+^) 12,51 with the ability of returning to the lymph nodes, as suggested by their expression of CCR7 and L-selectin 51,52. These cells are separated from the brain parenchyma by the *pia mater* and appear to influence and be influenced by events in the brain 9.

In addition to T cells, other immune components, such as B lymphocytes, granulocytes, macrophages, mast cells and dendritic cells, are present in the “meningeal structures” of the brain bathed by the CSF 12,8,53. Thus, suppression or deprivation of T cells can promote a pro-inflammatory phenotype in myeloid cells such as monocytes and granulocytes, with induction of high levels of IL-1β, IL-12 and TNF-α in the peripheral circulation and in the intracerebroventricular space, potentially triggering impairment of brain function and cognitive ability 49. The anti-inflammatory cytokine IL-4 is associated with cognitive ability by inducing the expression of BDNF by astrocytes, a factor fundamental for the maintenance of neurocognitive ability 12. Thus, IL-4 maintains the M2 phenotype of meningeal myeloid cells and regulates the expression of BDNF by neuroglial cells 12.

## Immune responses can trigger heterogeneous effects on brain functionality in experimental models

Stimulation of the immune system can have varying effects on cognitive function 54. Studies aimed at assessing the effect of immune stimuli on brain function report: i) the influence of neonatal vaccination on neuronal plasticity and cognitive function under normal physiological conditions in adulthood 55; ii) the impact of mitogenic and/or inflammatory stimulation of the immune system on the cognitive function of adult mice 56 and neuronal activation 57; iii) the effect of maternal immune stimulation on the neurocognitive performance of offspring 58; and iv) the increased risk of neurodegenerative disease development due to inflammatory process 59,60.

Immunization of C57BL/6 neonate mice with the BCG vaccine was shown to increase locomotivity as well as the distance travelled in the center of an open field and to improve spatial memory performance in the Morris water maze test. This was associated with increased peripheral and brain levels of IFN-γ and IL-4, neurogenesis and hippocampal BDNF, suggesting a beneficial immunomodulatory effect of immunization on cognitive function 55,61. Additionally, activation of an anti-inflammatory state of microglia and improvements of cognitive ability have been reported in C57BL/6 mice 62 and rats 54 immunized with BCG.

Also, the study of Brombacher et al. reinforced the importance of Th2 cytokines (IL-13 and IL-4) in the cognitive function of BALB/c mice challenged with the helminth *Nippostrongylus brasiliensis*, demonstrating the involvement of these cytokines in the stimulation of BDNF production by meningeal and hippocampal astrocytes 15.

IL-4 knockout mice and those in which IL-4-producing T cells are depleted exhibit meningeal myeloid cells with pro-inflammatory phenotype and are cognitively impaired 12. These data demonstrate a role for T cell derived IL-4 in the regulation of the pro- and anti-inflammatory phenotypes of meningeal myeloid cells and consequently in the expression of BDNF in the brain and the homeostasis of cognitive function 13.

Still on the beneficial effect of anti-inflammatory immune responses on the nervous system, it was reported that the non-inflammatory interleukin 17, IL-17, secreted by meningeal γδ T cells, is a promoter of short-term memory in mice 63.

Adverse effects of immune stimuli on cognitive function have also been demonstrated. Th1 cytokines activate neuroinflammatory processes and have a negative effect on brain function. Yang et al. 55 showed that neonate C57BL/6 mice immunized with the hepatitis B (HBV) vaccine performed poorly in the elevated plus maze and the Morris water maze. The mice had elevated levels of TNF-α, reduced levels of IFN-γ and demonstrated decreased neurogenesis and BDNF in the hippocampus at the eighth week of life. This suggests that neonatal HBV vaccination may result in detrimental behavioral effects in early adulthood. These data are corroborated by a study by Li et al. 54 who reported similar observations in rats. Inoculation of lipopolysaccharide (LPS) in C57BL/6 mice also increases levels of pro-inflammatory cytokines, mainly TNF-α and IL-1β, and induces infiltration of immune cells into the hippocampus, increased myeloperoxidase activity in the cortex and hippocampus and increased immunoreactivity of microglia and astrocytes, leading to neuronal damage and memory loss 64. Behavioral deficiencies related to memory, decreased locomotivity and anxiety-like behavior have been observed 10 months post LPS injection in Wistar rats 65. Even inoculation of minimum doses of LPS can cause spatial memory deficiencies in the Y-maze test in C57BL/6 mice 56.

More recently, experimental studies in mice have demonstrated that peripheral immune challenges with dextran sulfate sodium, which induce inflammatory bowel disease, can influence the activity of the insular cortex - a crucial cortical region involved in the perception of the body's physiological condition 66. Such an immune challenge is associated with activation of neurons in the insular cortex. Furthermore, neuronal reactivation was capable of reactivating the peripheral immune response even in the absence of immune challenges, leading to an increase in the percentage of leukocytes, the proportions of activated CD4 and CD8 T cells and dendritic cells, and the levels of TNF-α, IL-6 and IL-17 in intestinal compartments. These findings suggest that the insular cortex receives information from peripheral neurons responsive to signals about peripheral immune status and is an important brain region for the recovery of specific immune responses 57.

Murine models of maternal immune activation are widely used to study cognitive deficiencies (memory) and intrinsic changes involved in depressive- and anxiety-like behavior linked to neurodevelopmental alterations, and it is now known that stimuli that induce increased levels of pro-inflammatory cytokines in pregnant mice can cause neurocognitive deficits in the offspring. Although not demonstrated by Brabi et al. 67 at gestational day (GD) 17, the challenge of pregnant C57BL/6 mice at GD 9 with LPS causes anxiety and depression related behavior (revealed by elevated plus maze, open field, light-dark, forced swimming and tail suspension tasks) in the offspring at 8 and 10 weeks of age 68. In C57BL/6 pregnant mice immunized with Poly (I:C), increased levels of IL-1β were observed in the hippocampus of the offspring, a cytokine related to behavioral disorders 69. Under this immunization scenario, it has been reported that maternal pathway of Th17 cells and IL17A cytokine can induce autism-like phenotype in offspring 70. Additionally, Mueller et al*.* 58 reported deficiencies in working memory and changes in social behavior in the adult offspring at 9-12 weeks. The effects of maternal immune stimulation on the neurodevelopment of offspring in murine experimental models were recently systematized by Woods et al*.* 71.

One particular detrimental effect of maternal immune activation may be associated with exacerbation of Th2 cytokines levels. C57BL/6 mice desensitized with ovalbumin (OVA) present attenuated allergic asthma when challenged intranasally. Female C57BL/6 mice exposed to aerosolized OVA before and during pregnancy and their offspring showed social interaction deficiencies and increased object-burying behavior when compared to the offspring of mothers not exposed to OVA 72. Using this same model, it was shown that chronic OVA-induced asthma causes learning and memory deficiencies as assessed by the Morris water maze test 73. Similarly, neonatal overexposure to IL-4 is associated with neuroinflammation and cognitive impairment in C57BL/6 mice 74. Such negative effects of IL-4 on cognitive-behavioral performance were also observed following neonatal HBV vaccination 55.

Events characteristic of the inflammatory process such as induction of cytokines and activation of immune cells, as well as dysfunctions of the immune system can increase the risks of development of neurodegenerative diseases such as multiple sclerosis and Parkinson's disease 10,59,60. Some reports support the "theory of depression induced by cytokines", by Maes 75, demonstrating that depressive disorders, in the absence of somatic comorbidities, such as heart disease and obesity, may be associated with increased concentrations of pro-inflammatory cytokines such as TNF-α, IL-1β and IL-6 in the CNS and periphery 76-79. These inflammatory mediators may impact the cognitive functioning of individuals causing impairment of learning and memory, loss of visual and spatial perception abilities, loss of verbal and executive fluency, attention deficits, and inhibition of reaction, planning and problem solving - effects seen even in mild cases of depression 78.

As described earlier, peripheral immune challenges inducing pro-inflammatory immune responses may be associated with alterations in brain function, triggered by neurochemical and neuroinflammatory events that can negatively impact brain plasticity and cognitive ability. These changes include the reduction of important neurotrophins for cognition, such as BDNF, as well as decreased neurogenesis.

## *Plasmodium* infection can cause neurocognitive impairment in humans

Given the above, changes in physiological neuroimmune communication as a result of the immune response triggered by infectious diseases such as malaria, may be expected to result in cognitive changes.

Around 92% of malaria cases in the world are due to *P. falciparum*; 1 to 2% of which progress to CM, which causes approximately 80% of the deaths due to malaria 80. In humans, the main cellular event of CM is the sequestration of red blood cells, mostly parasitized, platelets and leukocytes in cerebral blood vessels, triggering microvasculature obstruction, inflammation and petechial hemorrhage in the brain and cerebellum 81. The onset of CM can be gradual or sudden and the condition can evolve from simple headache to deep coma within a couple of hours in children and non-immune adults. Adult patients with CM may present symptoms that mimic those of acute delirium, brain abscess, hypertensive encephalopathy, viral encephalitis, epilepsy, heat stroke, intoxication with drugs and poisons and bacterial, fungal or protozoan meningoencephalitis 82-84.

Approximately 15-25% of individuals diagnosed with CM die, even with prompt treatment with artemisinin derivatives that promote a very sharp decline in parasitemia, and survivors may have neurocognitive sequelae 85. CM is, therefore, a subject of great interest in malariology 22,32.

Neurocognitive sequelae of CM can occur in the short or long-term and may include ataxia, hemiparesis, monoparesis, hemiplegia, severe motor deficit, dysphasia, behavioral difficulties, severe learning difficulties and visual, auditory or linguistic impairment, and even epilepsy 86. In addition to these, hyperactivity and aggressive, self-injurious or destructive behaviors, anxiety and depression have also been reported 87-89.

Several studies indicate the existence of long-term neurocognitive sequelae that may lead to childhood cognitive impairments, especially (but not only) in Africa, where CM and its complications are more prevalent, particularly among children under five years old 22,32,80,86,88,90-95.

Severe neurological manifestations, such as coma 96, as well as levels of TNF-α, IL-6, IL-8 and granulocyte colony stimulating factor (G-CSF) 32, kynurenine and kynurenic acid 97 and angiopoietin-2 98 are increased in the CSF being associated with persistent neurocognitive sequelae of Ugandan child recovering from CM. High levels of Tau proteins in CSF and plasma are also associated with neurocognitive impairment related to attention, associative memory and working memory following CM 93,99. These proteins are equally associated with malaria retinopathy 94, and with dementia in Alzheimer's disease 100.

There is, furthermore, a clear relationship between episodes of malaria and reduced cognitive function associated with memory and learning in non-severe forms of malaria 18,95,101-104. In Sri Lanka, for example, where the most prevalent species were *P. falciparum* and *P. vivax*, children with a history of more than five episodes of non-severe malaria performed less well in writing, language and mathematics than those with a history of three or less episodes. Secondary factors such as socio-economic and nutritional status of the children were also considered, but the main factor influencing the academic performance of these children was the number of previous malaria episodes 101.

In Zambia, exposure of children under five years of age to *Plasmodium* infection has a negative effect on pre-school and educational development, indicating that exposure to the parasite exerts an impact not only on children's health, but on their cognitive development as well 103.

The first evidence of an association between non-severe malaria and learning impairments in Latin America was reported by Vitor-Silva et al. 102 who observed reduced performance in school tests (final average in Portuguese and mathematics) after at least one non-severe *P. falciparum* and/or *P. vivax* infection, in children in the Brazilian Amazon. Cognitive impairment, assessed by an indicator of intellectual function, Intelligence-IV (WPPSI-IV) which measures parameters such as verbal comprehension and working memory, was reported in children between 2 and 7 years old who had suffered at least one *P. vivax* malaria episode compared to children who had never had malaria 104. More recently it was reported that acute *P. vivax* malaria may be associated with long-term impairment of executive and cognitive functions in the elderly 105.

Evidence of neurocognitive impairment related to motor ability and fitness in the TEA-Ch test (Everyday Attention Test for Children) was found in individuals infected with *Plasmodium* but asymptomatic for malaria in the Republic of Yemen and Uganda, respectively, where *P. falciparum* is the most prevalent species 106.

In addition to the aforementioned sequelae, retinopathy - although rarely documented when compared to *P. falciparum* malaria 107 - is a possible sequela of non-severe *P. vivax* malaria.

## *Plasmodium* infection can cause neurocognitive impairment in mice

**Figure 2:**
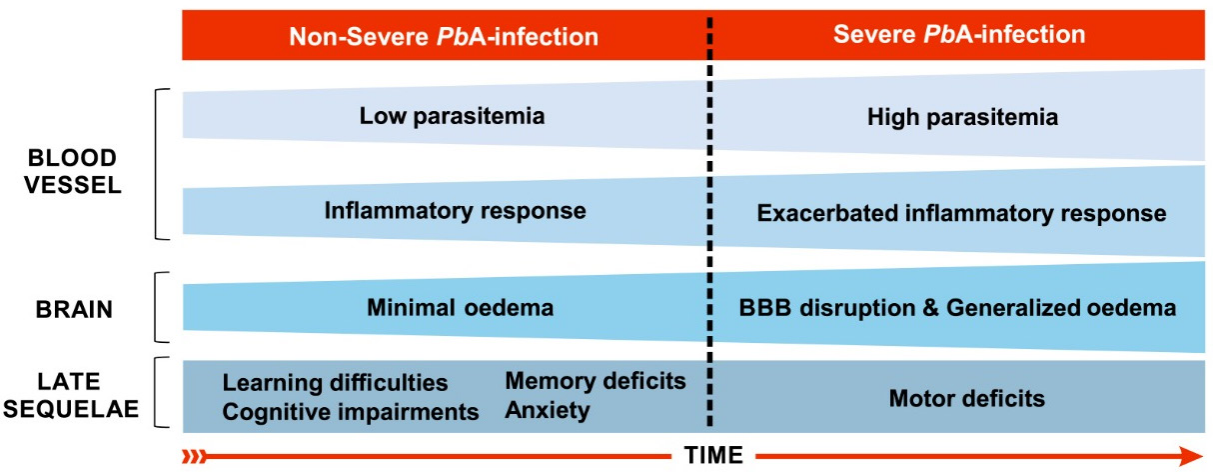
** Main features of severe and non-severe malaria in C57BL/6 mice infected by *Plasmodium berghei* ANKA**. On the fourth day of infection, when the disease can still be characterized as a non-severe malaria, low inflammatory response (few leukocytes adhered to blood vessels) in the brain are recorded, along with low parasitemia (about 2.5%) and oedema 108. Between the fifth and sixth days of infection, murine cerebral malaria may start to establish. On the fifth day, such events expand throughout the brain and an increase in parasitaemia occurs 108. Although parasitized red blood cells can also be observed in the brain microenvironment 109, there is a predominance of leukocyte accumulation, mainly T cells, in the cerebral microvasculature 81. Generalized and severe bleeding, elevated levels of edema and leukocyte adhesion throughout the brain, and damage to the BBB occur from the sixth day of infection on 108. Long-term cognitive and behavioral deficits, such as learning and memory difficulties and anxiety-like behavior, are observed as sequelae using this model of infection, including in mice treated the day before the onset of cerebral malaria establishment, the fourth day of infection 23,24. These sequelae are also evident after cerebral malaria along with motor system impairment 17,33.

In the murine experimental model of CM (ECM), involving the infection of C57BL/6 mice with the ANKA strain of *Plasmodium berghei* (*Pb*A), the evolution and establishment of ECM occurs between the fifth and sixth days of infection 108 and is characterized by the accumulation of leukocytes, mainly T cells and macrophages, in the cerebral microvasculature 81, although parasitized red blood cells may also be observed in the cerebral microenvironment 109. Consistent with CM in humans, hemorrhage and inflammation also occur with a consequent breakdown of the BBB (Fig. 2), causing brain damage in regions that are important for cognitive performance such as the cortex and hippocampus 110.

The initial neuropathogenesis of C57BL/6 mice infection with *Pb*A occurs in the olfactory bulb where the early expression of chemokines, especially CCL21, on the third day after infection, could explain the impaired smelling function recorded from the fourth day on, as assessed by the buried food test 111. Potter et al. 108 describe the kinetics of histopathological events associated with ECM development in this murine model. On the fourth day of infection low numbers of leukocytes are recorded adhering to vessels in the brain, along with low parasitaemia and minimal edema. On the fifth day of infection, such events appear throughout the whole brain and an increase of the parasitemia occurs. Generalized and severe hemorrhage, edema and adherence of leukocytes throughout the brain occur on the sixth day of infection.

The pathophysiological processes of ECM are dependent on the host's immune response. Howland et al. 112 considered CD8 T cells to be the main mediators of death in ECM. Approximately 90% of lymphocytes sequestered in the brain express CXCR3, associated with type 1 immune responses and inflammation, and mice deficient in this chemokine receptor have reduced traffic of CD8 T cells to the brain and 70-90% protection against the development of ECM. There is a strong association between the expression of this receptor and migration of T cells to the organs and the development of ECM 113.

In summary, recruitment of CD8 T cells to the brain results in increased expression of pro-inflammatory cytokines such as IFN-γ, TNF-α and chemokines that promote the activation of cerebral microvasculature endothelial cells and expression of receptors and ligands that favor leukocyte adhesion 114.

The neurological syndrome of ECM is characterized by paralysis, ataxia, convulsions, and coma 82. Animals that survive this syndrome experience neurocognitive sequelae such as motor impairments and memory-related disorders, apparent both immediately and 30-40 days after infection 17,22,33,95,115. Anxiety-like behavior has also been reported on the fifth day of infection 116, but may not represent a behavioral sequela, since animals were assessed during an active infection.

Although observed in the short or medium term after non-severe *P. falciparum* and *P. vivax* malaria, cognitive impairments have not been recorded in the experimental murine models considered standard for the study of non-severe malaria, such as BALB/c, C57BL/6 and Swiss mice infected by *Pb*A, *P. chabaudi chabaudi* and *P. yoelii* 17NL, respectively 17,116. These animals were evaluated using behavioral tasks such as open field and novel object recognition test, which analyze short-term and long-term memory.

More recently, however, de Sousa et al. 23 reported cognitive-behavioral impairments related to long-term memory and anxiety-like behavior late after a single episode of non-severe experimental malaria (Fig. 2), if the model susceptible to the development of ECM - C57BL/6 mice infected with *Pb*A - is used, with mice treated before the appearance of any sign of neurological impairment. These data suggest that the use of the *Pb*A infection model, with treatment prior to the appearance of the clinical signs of ECM, may create an experimental condition that somehow simulates the cases of non-severe *P. falciparum* human malaria that can potentially evolve to CM. The data of de Sousa et al. 23 also seem to indicate that even if signs of neurological impairment are not immediately measurable, malaria parasite infection may induce early changes in the physiology of the brain indirectly through the dissemination of inflammatory mediators released during systemic inflammation. For this reason, we have been using the expression “non-severe malaria” or even non-complicated malaria, instead of “non-cerebral malaria”, to describe the early events of *Pb*A infection in C57BL/6 mice 23,24,95.

Table S1 illustrates studies reporting behavioral changes studied in *Pb*A infected C57BL/6 mice, indicating the brain anatomic regions related to the neurocognitive impairment in the referred experimental model or found affected in the quoted study. The reported changes have been observed during infection (both severe and non-severe) and/or after the end of treatment, being of short or long-term duration.

## Re-establishment of neurocognitive function by immune responses in mice

Immunization procedures can reverse disease-associated neurocognitive deficiencies. Zuo et al. 117 observed a beneficial effect of BCG vaccination in the murine model of Alzheimer's Disease (AD). Immunization promoted an increase in levels of IL-4, IL-10, TGF-β1 and neurotrophic factors in the brain. This procedure did not decrease the concentration of β-amyloid (Aβ) in the brain, but it was able to induce a beneficial immunomodulatory effect by attenuating cognitive deficits as measured by the Morris water maze test. Using the same experimental model and behavioral assessment, Xing et al. 118 reported that immunization with a DNA vaccine (encoding ten tandem repeats of Aβ3-10 fused with mouse IL-4), performed when AD was already established in the mouse, reduced cerebral inflammation and attenuated cognitive impairment.

An increase in the Aβ peptide is seen in Down Syndrome together with cognitive difficulties. In the experimental model of segmental trisomy 16 119, Belichenko et al. 120 reported improvements in cognitive ability after immunization of adult Ts65Dn mice with the Aβ peptide. Immunization minimally reduced Aβ levels in the brain, but significantly restricted the atrophy of cholinergic neurons and improved short-term memory.

Anxiety-like behavior shares some characteristics with depressive behavior, such as a reduction of BDNF and impairment of neurogenesis in the hippocampus. Lewitus et al. 121 investigated the impact of immunization with a myelin-derived peptide in rats with depressive behavior and found that immunization restored levels of BDNF and neurogenesis in the hippocampus and attenuated anxious and depressive behavior.

Recently, de Sousa et al. 24 reported a beneficial modulatory effect of stimuli with immunogens able to induce type 2 immune responses having as one of its components the Td vaccine used in humans, on cognition of healthy adult C57BL/6 mice. These stimuli were able to reverse the cognitive-behavioral deficiencies characteristic of *Pb*A infection in which mice are chloroquine treated before the onset of ECM 23 (Fig. 1). This reversal effect of cognitive-behavioral damage was also observed when using only the Td vaccine as immunogen 122. These results offer an additional potential approach for improving cognition and for recovering cognitive behavioral damage caused by chronic or infectious diseases, including malaria, as we have recently proposed 123.

## Conclusions

The immune and nervous systems are interrelated cognitive systems that interact both in homeostasis and in abnormal conditions. Exogenous stimuli such as infectious agents and vaccines stimulate the immune system, modifying it structurally and operationally. Depending on the nature of the immune responses they trigger, stimuli can heterogeneously impact the functioning of the CNS and consequent cognitive behavioral responses. The studies discussed here demonstrate both the benefits and harm that may be exerted by the immune response on neurocognitive performance. Severe and even non-severe malaria can result in memory deficiencies and anxiety-like phenotypes in humans and mice. Immune-stimulation of *P. berghei* ANKA infected and treated mice can reverse these sequelae. Further studies on the interplay between the immune and nervous systems in infectious and chronic degenerative diseases and on the properties and composition of the immune stimuli capable of influencing neurocognitive parameters are needed. A better understanding of how these immunomodulatory agents operate to improve neurocognitive behavioral performance may open up a range of possibilities for the use of vaccines beyond their classic use as infectious disease prophylactics.

## Supplementary Material

Supplementary table S1.
