# Supplementary material for: Interplay Between the Immune and Nervous Cognitive Systems in Homeostasis and in Malaria
Source: Int J Biol Sci. 2023 Jun 28;19(11):3383–94. doi: 10.7150/ijbs.82556 (PMC10367562; doi:10.7150/ijbs.82556)
Supplement: Supplementary file 1 — Supplementary table S1. [file ijbsv19p3383s1.pdf]

Table 1. Behavioral alterations in *Plasmodium berghei* ANKA infected C57BL/6 mice model of experimental malaria

dpi	Paradigm	Parameter	Performance/deficit	Related neuroanatomic regions	OBS	REF
7	NORT	New object exploration	Short-term visual working memory deficit	Hippocampus#[$], cortex#, posterior parahippocampal region[$]*, perirhinal cortex[$]*, fornix#, thalamus#, midbrain#, cerebellum#	Behavior alterations in NORT at 5 dpi and locomotor activity in OFT at 5, 7 and 11 dpi were NS.	124
~15	OFT	Crossings and rearings	Long-term habituation memory deficit	Hippocampus#[$] (OFT, NORT), cerebral cortex#[$] (OFT), cerebellum#, dorsal hippocampus[$]* (IAT) entorhinal and parietal cortex[$]* (IAT) and/or amygdala[$]* (IAT)	Locomotor activity and exploration in OFT at 20 dpi and latency time in Continuous multiple trials step-down IAT at 15 dpi were NS.	17
	IAT	Latency test	Long-term aversive memory deficit			
	Continuous multiple trials step-down IAT	Training trials to reach the platform	Learning impairment			
	NORT	Index of recognition	Short and long-term recognition memory deficit			
~30	OFT	Crossings and rearings	Habituation memory deficit			
9	OFT	Grids crossed/min and rears	Lower activity	Brainstem#, medulla#, cortex#, hippocampus#, cerebellum#[$] (BBT)	Preference scores in NORT at ~56 dpi were NS with 45 min of RI. Grip strength in BBT at ~26 or ~56 dpi were NS. Slips and latency in BBT (1.8 cm beam) at ~56 dpi were NS, slips at ~26 dpi were NS and more latency at ~26 dpi was detected.	33
~26	OPT	Preference scores	Short-term spatial memory deficit			
	NORT	Preference scores	Short-term recognition memory deficit (45 min RI)			
	BBT	Slips and latency	Motor coordination deficit (1.2 cm beam)			
~56	OPT	Preference scores	Short-term spatial memory deficit			
	NORT	Preference scores	Short-term recognition memory deficit (120 min RI)			
	BBT	Slips and latency	Motor coordination deficit (1.2 cm beam)			
5	EPM	Open arm time and entries	Anxiety-like behavior	Brainstem#, cerebral cortex# and hippocampus#	Closed arms entries in EPM at 5 dpi were NS.	125
15	IAT	Latency	Short-term aversive memory deficit	Cerebral cortex#	-	126
5	Step-down IAT	Latency	Short-term aversive memory deficit	Hippocampus# and frontal cortex#	Latency 24h RI in Step-down IAT at ~5 dpi was NS. Crossings and rearings in OFT at 5 dpi were NS.	127
4	BFT	Time to find food	Loss of smell	Olfactory bulb#[$]	-	111

Table 1 (cont.). Behavioral alterations in *Plasmodium berghei* ANKA infected C57BL/6 mice model of experimental malaria

dpi	Paradigm	Parameter	Performance/deficit	Related neuroanatomic regions	OBS	REF
5	NORT	New object exploration	Working memory deficit	Hippocampus#[$], frontal cortex#[$]	Distance traveled in NORT at 5 dpi was NS.	128
18<br>5<br><br><br>17	EPM<br>OFT<br>NORT<br>MWM<br>NORT	Open arm time and entries<br>Distance traveled<br>Distance traveled<br>New object exploration<br>Time in the target quadrant<br>New object exploration	Anxiety-like behavior<br>Anxiety-like behavior<br>Sickness behavior<br>Long-term recognition memory deficits<br>Long-term spatial memory deficits<br>Long-term recognition memory deficits	Prefrontal cortex#, Hippocampus#	Crossings in OFT at 5 dpi, enclosed arms entries in EPM at 18 dpi, distance traveled in NORT at 17 dpi were NS.	115
6, ~21 and 52	NORT	New object exploration	Working memory deficit	Brainstem#, cerebellum#, hippocampus#, cerebral cortex#	-	110
5	NORT<br>Y Maze	New object exploration<br>New arm exploration	Short-term memory deficit<br>Spatial memory deficit	Hippocampus#[$], frontal cortex#, subventricular zone#, cerebellum#, olfactory bulb#, corpus callosum#	-	22
~25	NORT<br>FST	New object exploration<br>Immobility	Long-term recognition memory deficit<br>Depressive-like behavior	Frontal cortex#, hippocampus#	Latency in training and test sessions of Step-Down IAT at ~25 dpi were NS. Distance traveled in NORT at ~25 dpi was NS. Open arms time and entries in EPM at ~25 dpi were NS. Time in center in OFT at ~25 dpi were NS.	129
~45	MB<br>NB	Marble buried<br>Nest score	Neophobia or compulsive-like behavior<br>Spontaneous behavior impairment	Hippocampus#[$], cerebral cortex#	Crossings, rearings and time in center in OFT at ~45 dpi were NS. Latency to fall in Rotarod at ~45 dpi were NS. Consumption in sucrose preference at ~45 dpi were NS.	130
~74	OFT<br><br>NORT<br>Y Maze<br>LDT	Distance traveled<br>Centre distance<br>New object exploration<br>Time in each arm<br>Time in light zone	Short-term habituation deficit (1st and 3rd min)<br>Anxiety-like behavior<br>Long-term recognition memory deficit<br>Different temporal pattern suggesting anxiety-like behavior<br>Anxiety-like behavior	NA	Total distance traveled and time in center zone in OFT at ~74 dpi were NS. Percentage of spontaneous arm alternance in Y-Maze at ~74 dpi was NS. Transitions in LDT at ~74 dpi were NS.	23

Table 1 (cont). Behavioral alterations in *Plasmodium berghei* ANKA infected C57BL/6 mice model of experimental malaria

dpi	Paradigm	Parameter	Performance/deficit	Related neuroanatomic regions	OBS	REF
5 and 15	TST<br>FST	Immobility	Depressive-like behavior	Cortex#$^{\$}$	-	131
6	OFT	Crossings<br>Groomings<br>Lifting	Locomotor and exploratory behavior deficits	Cortex#	-	132
~88	OFT<br>NORT<br>LDT	Centre distance<br>New object exploration<br>Time in light zone	Anxiety-like behavior<br>Long-term memory deficit<br>Anxiety-like behavior	Hippocampus$^{\$}$*	Distance traveled in OFT at ~88 dpi was NS.	24
~12	Y Maze<br>MWM	Alternations and successful alternations (%)<br>Escape latency and distance to reach the platform	Memory deficit<br>Memory deficits	Cerebellum#, midbrain#, olfactory bulb#, cortex#	-	133
6-8<br>~31-53	CT<br>ART<br>BBT<br>BMT<br>T maze<br>One-trial NORT	Rears<br>Time of contact<br>Time of removal<br>Time to traverse the beam and foot slips<br>Primary latency and errors<br>Correct alternations and side preference rate<br>New object exploration and discrimination index	Spontaneous activity impairment<br>Sensorimotor activity deficit<br>Motor coordination deficits<br>Spatial memory deficits<br>Short-term memory deficits<br>Recognition memory deficits	Nigrostriatal$^{\$}$* (CT and ART), hippocampus#$^{\$}$ (T maze, BMT, NORT)	Rears, times of contact and removal in CT and ART at ~35 dpi were NS.	134
~22 and ~155	NORT<br>LDT	New object exploration<br>Time in light zone	Long-term memory<br>Anxiety-like behavior	Hippocampus$^{\$}$*	Distance traveled and centre distance in OFT at ~22 and ~155 dpi were NS. Time in center zone in OFT at ~22, ~87 and ~155 dpi were NS (except anxiety-like behavior in larger	122

					arena at ~155 dpi). Transitions in LDT at ~22, ~87 and ~155 dpi were NS.	

dpi: days post-infection; OBS: observations; REF: reference; ~: approximately; RI: retention interval; cm: centimeters; min: minutes; NS: not significant; NA: not available; # region analyzed in the study during infection; * region suggested in discussion; [$] neuroanatomic region related to the function; - : no remark; NORT: novel object recognition task; OFT: open field test; IAT: inhibitory avoidance task; OPT: object placement test; BBT: balance beam test; EPM: elevated plus maze; BFT: buried food test; MWM: morris water maze; FST: forced swim test; MB: marble burying; NB: nest building; LDT: Light/Dark task; TST: tail suspension test; CT: cylinder test; ART: adhesive removal test; BMT: barnes maze test.
